# Perchlorate Exposure Reduces Primordial Germ Cell Number in Female Threespine Stickleback

**DOI:** 10.1371/journal.pone.0157792

**Published:** 2016-07-06

**Authors:** Ann M. Petersen, Nathanial C. Earp, Mandy E. Redmond, John H. Postlethwait, Frank A. von Hippel, C. Loren Buck, William A. Cresko

**Affiliations:** 1 Institute of Ecology and Evolution, Department of Biology, University of Oregon, Eugene, Oregon, 97403, United States of America; 2 Institute of Neuroscience, Department of Biology, University of Oregon, Eugene, Oregon, 97403, United States of America; 3 Department of Biological Sciences, Northern Arizona University, Flagstaff, Arizona, 86001, United States of America; 4 Department of Biological Sciences & Center for Bioengineering Innovation, Northern Arizona University, Flagstaff, Arizona, 86001, United States of America; 5 Department of Integrative Biology, Oregon State University Cascades, Bend, Oregon 97703, United States of America; University of Rouen, France, FRANCE

## Abstract

Perchlorate is a common aquatic contaminant that has long been known to affect thyroid function in vertebrates, including humans. More recently perchlorate has been shown to affect primordial sexual differentiation in the aquatic model fishes zebrafish and threespine stickleback, but the mechanism has been unclear. Stickleback exposed to perchlorate from fertilization have increased androgen levels in the embryo and disrupted reproductive morphologies as adults, suggesting that perchlorate could disrupt the earliest stages of primordial sexual differentiation when primordial germ cells (PGCs) begin to form the gonad. Female stickleback have three to four times the number of PGCs as males during the first weeks of development. We hypothesized that perchlorate exposure affects primordial sexual differentiation by reducing the number of germ cells in the gonad during an important window of stickleback sex determination at 14–18 days post fertilization (dpf). We tested this hypothesis by quantifying the number of PGCs at 16 dpf in control and 100 mg/L perchlorate-treated male and female stickleback. Perchlorate exposure from the time of fertilization resulted in significantly reduced PGC number only in genotypic females, suggesting that the masculinizing effects of perchlorate observed in adult stickleback may result from early changes to the number of PGCs at a time critical for sex determination. To our knowledge, this is the first evidence of a connection between an endocrine disruptor and reduction in PGC number prior to the first meiosis during sex determination. These findings suggest that a mode of action of perchlorate on adult reproductive phenotypes in vertebrates, including humans, such as altered fecundity and sex reversal or intersex gonads, may stem from early changes to germ cell development.

## Introduction

Perchlorate is a common aquatic contaminant that has been shown to affect thyroid function by competitively inhibiting iodide uptake at the sodium-iodide symporter [1]. This small molecule has been linked to a variety of developmental abnormalities associated with hypothyroidism in humans, including reduced cognitive function [2, 3]. Experimental exposure to perchlorate alters development and morphology of thyroid follicles in model organisms such as fish [4–6] and mice [7]. Perchlorate exposure in teleost fishes leads to a wide array of altered phenotypes, some of which suggest organizational effects during development in addition to activational effects on the Hypothalamic-Pituitary-Thyroid (HPT) axis [4, 8–11]. Perchlorate exposure has negative effects on health and survivorship of vertebrates at environmentally relevant concentrations, usually in the range of parts per billion [12, 13], and in some parts of Texas, USA, levels have been measured in surface waters in the parts per million range [14].

In addition to its effects on thyroid development and function, perchlorate also appears to act through an unknown mechanism to alter gonad development and sex determination in teleost fishes, a finding not predicted to occur solely *via* thyroid disruption [4, 6, 8, 11, 15]. Changes to reproductive development include increased androgen levels in larvae and adults, increased spermatogenesis, decreased oocyte maturation [6], and disruption of reproductive behaviors [16]. Perchlorate masculinizes the gonad in male and female threespine stickleback (*Gasterosteus aculeatus*; hereafter ‘stickleback’), in addition to increasing the gonadal-somatic index in male stickleback [6, 8]. In some cases, perchlorate exposure causes genotypically female stickleback to develop into functional hermaphrodites [8]. Exposure to perchlorate in the first 42 days post fertilization (dpf) causes abnormalities in development of thyroid and reproductive systems in adult stickleback, suggesting early critical windows of development in which this chemical exerts lasting effects into adulthood [4, 10]. Perchlorate also affects sexual differentiation in zebrafish (*Danio rerio*), a species in which different strains are reported to have varying or no genetic bases for sex determination [17–19]. In contrast to our findings in stickleback, exposure of zebrafish to perchlorate during early development skews the sex ratio towards female [15], suggesting some species-specific effects of perchlorate on sexual development.

Although perchlorate affects sexual differentiation in the stickleback and zebrafish teleost models, the mechanisms underlying the observed effects are unknown. In particular, it is not clear how perchlorate might affect the earliest stages of sexual differentiation, such as initial gonad organogenesis. In this study, we examine whether the earliest known morphological marker of sex determination in stickleback, primordial germ cell (PGC) number, is affected by perchlorate exposure beginning at fertilization. Specifically, we examine whether perchlorate alters sex determination and reproductive development by affecting proliferation or survival of the PGCs during early development. PGC development is one of the first tractable morphological events in vertebrates, occurring by either preformation or epigenesis, with both modes of specification found within teleosts [20].

Once they are specified, germ cells undergo several rounds of mitosis [21–23] and then migrate to the site of the presumptive gonad. At this point, the gonad is bipotential, with the capacity to become either ovary or testis. In many teleosts, including stickleback, the germ cells then begin to mitotically divide at a sex specific rate [24]. The underlying genetic signals and morphological changes that transform the bipotential gonads into either ovaries or testes vary widely among species [25]. The first sex-specific event in the developing gonad of many species is a second wave of several rounds of mitosis.

Genotypic female stickleback exhibit a larger and earlier second wave of mitosis than males at approximately 15 dpf, which results in three to four times more germ cells [24]. Between 15 and 18 dpf, germ cells in females undergo a round of apoptosis, with females having five times the number of apoptotic germ cells as males [24]. Following meiosis at approximately 18 to 20 dpf, females still have more germ cells than males, although the difference between the sexes is not as dramatic due to the large pre-meiotic apoptosis in the female germ cell population that occurs in the time period leading up to 18 dpf. In addition, the timing of the second wave of mitosis and meiosis of germ cells is earlier in female mammals such as mice [26] and humans [27].

This program of germ cell development is common to differentiation during sex determination of the primordial germ line in many teleosts including zebrafish [28–30], as well as in some other vertebrates [27] and represents one of the earliest sex specific, morphologically identifiable events in vertebrate development. For example, PGCs of male mouse embryos enter mitotic arrest at 13.5 dpf, whereas female PGCs continue to proliferate for an additional 24 hours before entering arrest [25, 31]. Intriguingly, when germ cells are ablated in medaka and zebrafish (strain AB) embryos, the modified fish all subsequently develop as males [29, 32, 33] suggesting that a lack of pre-oocyte signaling to the surrounding somatic tissue leads to a testis fate. Transplanting additional germ cells to the gonad reveals that a certain threshold of germ cells must be reached in zebrafish to maintain female identity and for the gonad to develop into an ovary [33]. Mutant zebrafish that develop and reproduce as females can, after germ cell loss at five months due to a mutation in *nanos3*, become males capable of forming functional sperm [34]. The current hypothesis for this outcome in zebrafish is that meiotic germ cells signal the gonadal somatic cells to maintain expression of aromatase, which converts testosterone to estrogen, causing the gonad to develop into an ovary and the fish to become a female [29, 30, 34, 35]. Reduction in germ cells due to the *nanos3* mutation therefore leads to a sex reversal from female to male in these zebrafish. Collectively, these data suggest a hypothesis that in vertebrates timing and magnitude of germ cell mitosis and meiosis, and therefore germ cell number, are critical drivers of reproductive development, including sex determination.

Here we use the stickleback fish model to address a potential cellular mechanism by which perchlorate influences reproductive development in vertebrates. We hypothesize that perchlorate exposure affects germ cell proliferation. We predict that the previously documented masculinizing effects of perchlorate exposure early in stickleback development would manifest as fewer germ cells in females, with numbers more similar to those observed in genotypic male stickleback [24]. We tested this hypothesis by exposing stickleback to environmentally relevant concentrations of perchlorate (100 mg/L) [36, 37], and counted premeiotic, meiotic, and total number of germ cells at 16 dpf, the earliest sex-specific difference in morphology in the stickleback gonad [24]. We exposed embryos to perchlorate-treated water from the time of fertilization until 16 dpf and counted germ cell number to test if perchlorate changes the magnitude of the second wave of mitosis. We found that perchlorate exposure decreases primordial germ cell number in female stickleback at 16 dpf, a time when genetic signaling cascades involving germ cell number are important to sex determination. Our findings suggest a cellular mechanism for the patterns of gonadal dysgenesis that we previously reported in perchlorate treated juveniles and adults.

## Methods

### Stickleback Husbandry

Anadromous stickleback were collected from Eel Creek, Oregon (43°35′N; 124°11′W) for parental stock to develop a laboratory line. These collections were covered by Oregon Scientific Take Permit # OR2007-3495, issued by the Oregon Department of Fish and Wildlife. The district biologist was contacted and approved of this sampling. No endangered or protected species were encountered during this sampling. However, the limited take of endangered salmonids (if encountered) was covered under the permit mentioned above. All collection, embryo production, and lab rearing methods were approved by the University of Oregon Institutional Animal Care and Use Committee, Protocol # 10–16. Stickleback were captured using minnow traps, and embryos were created by combining eggs with macerated testes in a Petri dish. Embryos were then transferred back to the lab and reared for multiple generations. All collection, embryo production, and lab rearing methods were approved by the University of Oregon Institutional Animal Care and Use Committee, Protocol # 10–16. All wild fish sampling procedures were proposed and approved upon applying and being rewarded with the sampling permit. Fish used in this study were the fourth and fifth generation of this line and were fertilized, hatched, and reared using standard crossing protocols [38]. At the onset of this study, fertilized, lab-reared clutches were divided evenly between control and perchlorate-exposure conditions to remove any effects of genetic background. Embryos and fry of all ages were maintained in 6 ppt Instant Ocean (Aquarium Systems, Mentor, OH) added to reverse osmosis water at a constant temperature of 20°C. Embryos and fry were raised under 12:12 light:dark cycle in glass 150 mm Petri dishes at a density of no more than 50 individuals/Petri dish (approximately 100 mL embryo medium), with daily water changes and removal of any dead or sick individuals. Sodium perchlorate hydrate (Sigma Aldrich, St. Louis, MO) was added to perchlorate-exposure dishes at a concentration of 100 mg/L (ppm), a concentration previously found to cause reproductive abnormalities in stickleback [6]. Both control and perchlorate treated water were changed daily until the end of the experiment (16dpf). After hatching (around 6 to 7 dpf), fry were fed live *Artemia* spp. (Inve Aquaculture, Inc., Salt Lake City, UT). Animal care, experimental methods, and euthanasia protocols were approved by the University of Oregon Institutional Animal Care and Use Committee, Protocol # 10-16R.

### Histology preparation

Thirty stickleback were euthanized at 16 dpf with neutral pH MS222 (Sigma-Aldrich, St. Louis, MO) and immediately fixed in Bouin’s solution and then placed at 4°C for 48 h. After Bouin’s fixation, fish were serially dehydrated in ETOH washes of increasing concentration, and then stored in 70% ETOH at 4°C. Samples were subsequently paraffin-embedded and sectioned sagittally through the gonad-containing portion of the abdomen to visualize the gonads at 8 μm resolution, and stained with hematoxylin and eosin (H&E). The University of Oregon Histology and Genetic Modification (HGeM) Core Facility performed sectioning and staining.

### Meiotic and premeiotic cell counts

**Fig 1:**
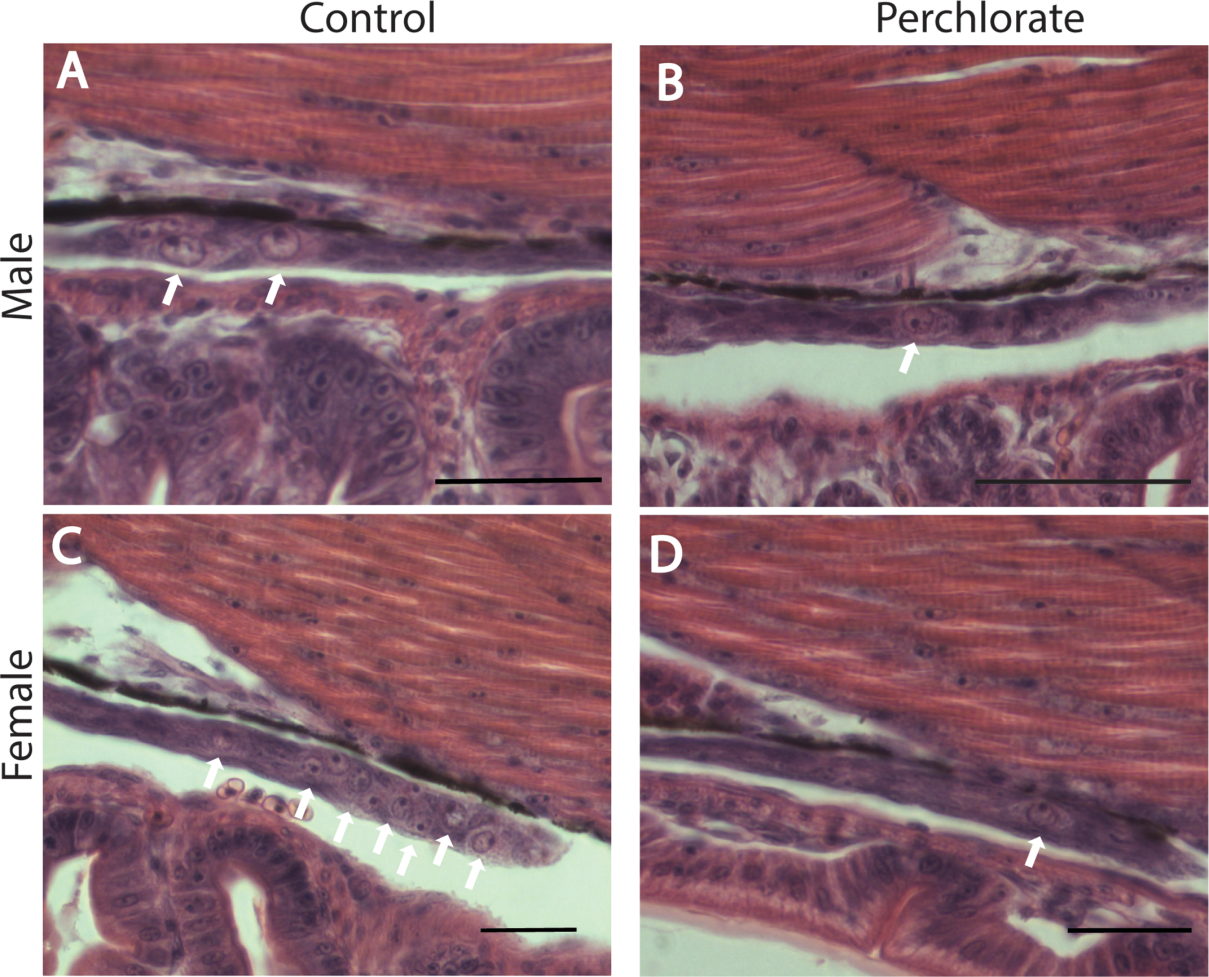
Hematoxylin and eosin staining of representative sagittal sections through the developing gonad in 16 dpf stickleback. Scale bars are 100 microns. White arrows denote location of PGCs within the gonad. At 16 dpf, number of PGCs in control male stickleback (A) were not different than in male stickleback raised in 100 mg/L perchlorate (B). At 16 dpf, however, control female stickleback (C) had more PGCs than females raised in 100 mg/L perchlorate (D).

Sagittal sections covered one to five slides (Starfrost) depending upon the size of the fry. Slides containing a 16 dpf fish resulted in approximately 30 sections. In 8 μm thick sections, it is possible to see multiple layers of cells in different focal planes. We counted PGCs at 400-600X under bright field illumination on every section that had visible gonad using a Nikon Eclipse Ti. PGCs were distinguishable by their size, shape, and location within the developing gonad (Fig 1). The entire gonad was sectioned into 10–37 sections, depending on the size of the gonad and the age of the fish. Only PGCs over 5 μm wide were counted and then classified as meiotic or premeiotic, to avoid recounting the same PGC that might span two sections, since the PGCs are spheres and therefore will be smaller than maximum width in consecutive sections. Premeiotic cells were distinguished from meiotic cells using the criteria of Lewis et al. (2008). Specifically, premeiotic cells were identified by size—premeiotic cells are typically twice the size—and by the presence of an un-condensed nucleus with an irregular nuclear membrane. PGC counts were tallied and analyzed based on treatment and sex genotype.

### Sex genotyping protocol

A standard sex genotyping protocol for stickleback was followed as described previously [39, 40]. Briefly, caudal fin was digested and DNA extracted using Qiagen DNAeasy kits. Primers described by Griffiths et al. 2000 (Primer 1 Forward 5’CTTCTTTCCTCACCATACTCA, Primer 1 Reverse 5’AGATGACGGGTTGATAAACAG) were used to amplify sex specific regions of DNA, and the resulting bands were visualized using standard gel electrophoresis protocols.

### Statistical Analysis

Analyses of all data were performed using JMP PRO software (2013 SAS Institute, Cary, NC). Means presented are +/- 1 SE. Comparisons of means for PGC number were analyzed using a two factor factorial analysis of variance (ANOVA) (effect of perchlorate treatment and sex on germ cell number), followed by Tukey’s HSD post-hoc analysis of means for significant treatment effects, after testing for statistical assumptions. Critical α = 0.05.

## Results

Pathohistological analysis of serially sectioned abdomens of 16 dpf stickleback revealed easily discernable PGCs in control males (Fig 1A) and females (Fig 1C) that were located in the cavity between the gut and the dorsal skeletal muscle. Female stickleback of this age appeared to have more PGCs in the bipotential gonad than males of the same age (Fig 1A and 1C). For stickleback raised in 100 mg/L perchlorate, females and males appeared to have approximately equal numbers of PGCs in the gonad (Fig 1B and 1D). The histological analysis did not reveal any other gross differences in the developing gonad at these ages between males and females, or between treated and control fish (Fig 1).

**Fig 2:**
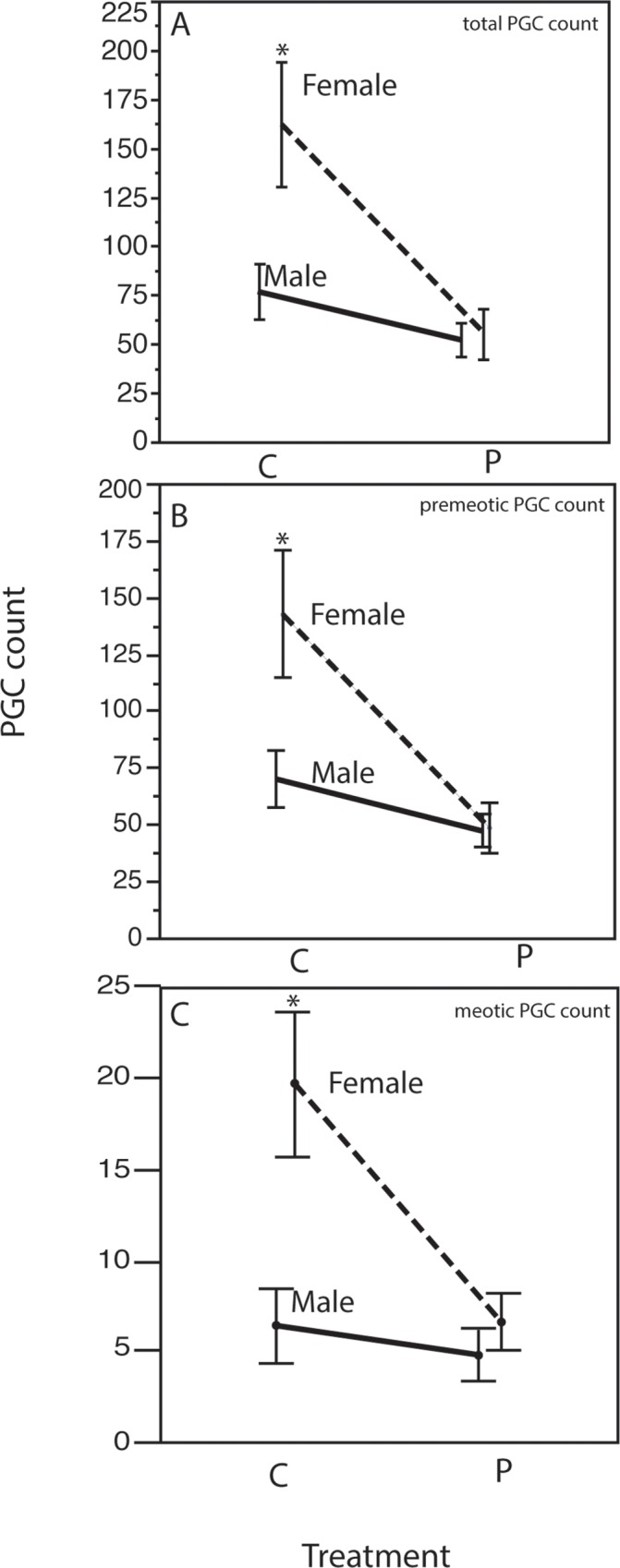
**Number of PGCs (+/-1SE) in 16 dpf genotypic male (solid lines) and female (dotted lines) stickleback, with interaction plots of the total number of PGCs (A), premeiotic PGCs (B), and meiotic cells (C).** Asterisk represents significantly different PGC counts (Two-Way ANOVA, Tukey's Post-Hoc Analysis, P< 0.05). n = 12–14.

To confirm histological observations, we counted the number of PGCs in entire gonads. Control 16 dpf males had a mean of 77 +/- 13.9 SE PGCs in the gonad, while control females had a mean of 163 +/- 31.8 SE PGCs (Fig 2A, S1 Data). A two-factor factorial ANOVA overall model effect confirmed a significant effect on PGC number for both sex (F_3, 22_ = 2.6, P = 0.0165) and perchlorate treatment (F_3, 22_ = 3.88, P = 0.0008), and a significant interaction effect of the two levels (F_3, 22_ = 7.23, P = 0.0015). Females exposed to perchlorate from fertilization to 16 dpf had significantly fewer (1/3 as many) total PGCs as control females, but perchlorate did not affect PGC number in males, explaining the interaction between perchlorate treatment and sex factors in the ANOVA model (Fig 2, S1 Data).

We categorized germ cells as either premeiotic or meiotic. In control female 16 dpf stickleback, a mean of 142.8 PGCs (88% of total) were premeiotic, while 12% had entered or completed meiosis (Fig 2B, S1 Data). In control male 16 dpf stickleback, a mean of 70.5 PGCs (92% of total) were premeiotic, with the remaining 8% meiotic (Fig 2C, S1 Data). The percent of PGCs that were premeiotic did not change with perchlorate treatment. In perchlorate treated 16 dpf females, a mean of 48.8 PGCs (88%) were premeiotic, and in perchlorate treated 16 dpf males 91% were premeiotic. At 16 dpf, perchlorate treatment results in a significant decline in both premeiotic (F_3, 22_ = 7.07, P = 0.0017) and meiotic (F_3, 22_ = 6.98, P = 0.0018) germ cell number only in females.

## Discussion

Stickleback are an established model system for testing the effects of endocrine disrupting chemicals relevant to human health [41, 42]. Using this model fish we found that perchlorate significantly reduces PGC number in female stickleback at 16 dpf, an important time in gonad development and possibly sex determination in this species [24]. The reduction in PGC number in genotypic females caused by perchlorate may explain how it masculinizes them, an effect that appears to have lasting consequences to the health and development of the adult fish. Our findings are one of the first reports of an endocrine disrupting contaminant affecting germ cell number in a developing vertebrate, an effect that might occur due to exposure to other contaminants as well. Our findings also underscore the utility of this model fish for ecotoxicology studies, and our results are likely extendible to other vertebrates. PGC number is the first tractable sex specific morphology in the developing gonad of many vertebrates [24, 29, 43, 44] and genetic signaling cascades from the larger number of meiotic germ cells in genotypic females drives female-specific cell fate in many teleosts [29, 30, 45] but interestingly not goldfish [46]. A gene important to male medaka sex determination, *Dmrt1bY*, inhibits germ cell proliferation, further suggesting that germ cell number during gonad development is closely linked to the sex determination process [43, 47].

The absolute numbers of PGCs we count differ from the findings of Lewis et al. (2008), which also examined PGC development in stickleback. In addition, we identify meiotic germ cells in females at 16dpf, as well as a few (5 or less on average) in males at this age (Fig 2). Lewis et al.(23)did not identify meiotic cells in either sex until 18dpf, although in that study no measurements were taken between 15dpf and 18dpf, making direct comparison impossible. In the current study, we examined an anadromous population of stickleback from Eel Creek in Oregon, while Lewis et al. (23 examined two Alaskan populations of stickleback from Rabbit Slough (anadromous) and Bear Paw Lake (resident freshwater). In the anadromous Alaskan population, Lewis et al. [24] counted up to ten times more PGCs in similarly aged fish. However, the resident freshwater population Lewis et al. [24] had PGC numbers more closely matched to the numbers we report here. The similarities and differences in the findings of these two studies raise the interesting possibility of population-specific patterns of germ cell development. Some plausible reasons for the differences between the studies include our use of a conservative quantification rubric to avoid counting the same cell twice, population level differences in developmental trajectories, and environmental factors such as temperature, salinity, and food availability causing different developmental patterns. A multi-population study of patterns of germ cell development across stickleback ecotypes would provide the necessary data to answer these questions. Population-level differences could also be exploited in genetic mapping studies to identify additional genes and pathways important for germ cell development, further underscoring the useful role for threespine stickleback as an evolutionary mutant model [48] for biomedical studies.

Our findings suggest that environmental perchlorate exposure early in life may be interfering with reproductive development during sex determination in exposed vertebrates. Germ cell number in carp has also been demonstrated to be affected by exposure to an estrogen mimic [49], but our data are the first to demonstrate an effect of a non-steroidal compound that is commonly found in drinking water sources. Early embryonic exposure to some pollutants causes lasting effects on adult reproductive morphology or performance in vertebrates [50–52] in addition to cancers [53–55] and other pathologies such as immunotoxic effects in the head kidney [56]. Some of these effects are transgenerational [55, 57], and an exciting area of research is on epigenetic mechanisms of inheritance of environmental exposure to pollutants. Perchlorate is well documented to disrupt the function of the thyroid with consequences to the hypothalamic-pituitary-thyroid axis [58–60], and perchlorate impacts health and development in ways that go beyond those easily interpretable as mediated *via* the thyroid. Many pollutants alter reproductive development, including ovarian follicle formation in mice [61], oocyte development in white suckers (*Catostomus commersoni)* [62], and spermatogenesis in stickleback [6]. The current study is one of the first to examine whether a known endocrine disruptor impacts reproductive development by affecting proliferation or survival of the PGCs prior to meiosis.

Although our data show that perchlorate clearly affects PGC number at 16 dpf in female stickleback (Figs 1 and 2), the molecular and physiological mechanisms are as yet unknown. Many pollutants, including perchlorate, cause adult gonad dysgenesis and pathology via effects on hormonal systems. Endocrine disruption by exogenous pollutants can alter gonad size [11, 12, 15], gonad shape [63], and reproductive development and behavior [8, 62]. We previously found that perchlorate masculinizes both adult male and female stickleback[6, 8], leading to the hypothesis that perchlorate can have androgenic effects. In a recent study [6], we found a significant effect of a range of doses of perchlorate on embryonic, larval, and juvenile levels of 11-ketotestosterone (11-KT). 11-KT is a key androgen in teleost fishes for stimulating male reproductive physiology [64, 65], and is synthesized from testosterone. Changes in embryonic 11-KT levels occurred within hours of perchlorate exposure [6]. Changes in the steroid environment in the embryo based on perchlorate exposure is therefore a possible mechanistic explanation for our finding in the current study that PGC number is reduced in perchlorate-treated females, but not in males. In tilapia, steroid-producing cells congregate near the differentiating germ cells, suggesting that steroids perform a role in the process of germ cell differentiation [66]. Androgens contribute to germ cell determination and follicular atresia in mice [67, 68]. Exposure of mice to environmental estrogens increases the occurrence of follicles containing multiple oocytes, which is a marker of disrupted germ cell development [61].

These results are consistent with the hypothesis that perchlorate disrupts PGC development by altering embryonic steroid levels (Fig 2). Perchlorate could reduce total PGC number in females by either reducing proliferation (mitosis) or by increasing the rate of cell death (apoptosis). Future studies should test these hypotheses using molecular markers of proliferation and apoptosis in perchlorate-treated and control fish. Quantifying PGC number across a span of development would be an important experiment to address these possibilities. Critical time periods of exposure have been detected for endocrine disrupting compounds, usually early in embryonic development [53, 69]. Chemical exposure is thought to explain numerous adult-onset human diseases [70, 71]. By 16 dpf, the second round of PGC mitosis has already occurred in stickleback, and females have measurably more PGCs than males. By 18 dpf, PGCs in control stickleback are entering into meiosis and differentiating into male or female reproductive cells, and between 16 and 18 dpf female stickleback experience a 5-fold increase in apoptotic cells, reducing germ cell number to a level close to that of males [24]. Our data suggest that perchlorate exerts effects on early stages of germ cell development, resulting in reproductive pathologies in adults [6, 10, 12].

## Conclusions

Perchlorate is a widespread aquatic contaminant in certain countries such as the United States, where nearly every person has measurable levels of perchlorate in their urine [72]. Here we demonstrate that perchlorate has effects on reproductive development by significantly reducing PGC number in female stickleback at 16 dpf, an important period of germ cell development and proliferation. Future studies should determine the mechanism by which perchlorate reduces PGC number, and whether this effect is widespread across vertebrates and therefore has implications for wildlife reproductive health. Because of the conservation of many aspects of germ cell production across vertebrates, our findings may also have significant implications for human health. In humans, disruption of endocrine function during early developmental windows can cause adult-onset diseases [73]. Our findings support the ‘developmental origins of health and disease hypothesis’ (DOHaD), which suggests that some adult human diseases arise from prenatal exposure to endocrine disrupting compounds [70, 74]. Our findings are likely applicable to the wide array of vertebrates in which germ cell number is an important early indicator of sex determination, including other teleosts and mammals. Environmental contaminants can cause primordial sexual dysgenesis in humans and may contribute to widespread diseases such as polycystic ovarian syndrome and breast cancer [74, 75]. Studies of model organisms such as stickleback have the potential to help in understanding–and eventually mitigating–these effects, by offering insights into mechanisms of action of contaminants that lead to pathology.

## Supporting Information

S1 Data Supplemental raw data. Raw counts of primordial germ cells in 16dpf threespine stickleback from Oregon.
(XLSX)
